# Sleep characteristics of persons with chronic fatigue syndrome and non-fatigued controls: results from a population-based study

**DOI:** 10.1186/1471-2377-6-41

**Published:** 2006-11-16

**Authors:** William C Reeves, Christine Heim, Elizabeth M Maloney, Laura Solomon Youngblood, Elizabeth R Unger, Michael J Decker, James F Jones, David B Rye

**Affiliations:** 1 Viral Exanthems & Herpesvirus Branch, Division of Viral & Rickettsial Diseases, National Center for Infectious Diseases, Centers for Disease Control & Prevention, Atlanta, GA, USA; 2 Department of Psychiatry and Behavioral Sciences, Emory University School of Medicine, Atlanta, GA, USA; 3 Dept. of Neurology, Emory University School of Medicine, Atlanta, GA, USA

## Abstract

**Background:**

The etiology and pathophysiology of chronic fatigue syndrome (CFS) remain inchoate. Attempts to elucidate the pathophysiology must consider sleep physiology, as unrefreshing sleep is the most commonly reported of the 8 case-defining symptoms of CFS. Although published studies have consistently reported inefficient sleep and documented a variable occurrence of previously undiagnosed primary sleep disorders, they have not identified characteristic disturbances in sleep architecture or a distinctive pattern of polysomnographic abnormalities associated with CFS.

**Methods:**

This study recruited CFS cases and non-fatigued controls from a population based study of CFS in Wichita, Kansas. Participants spent two nights in the research unit of a local hospital and underwent overnight polysomnographic and daytime multiple sleep latency testing in order to characterize sleep architecture.

**Results:**

Approximately 18% of persons with CFS and 7% of asymptomatic controls were diagnosed with severe primary sleep disorders and were excluded from further analysis. These rates were not significantly different. Persons with CFS had a significantly higher mean frequency of obstructive apnea per hour (p = .003); however, the difference was not clinically meaningful. Other characteristics of sleep architecture did not differ between persons with CFS and controls.

**Conclusion:**

Although disordered breathing during sleep may be associated with CFS, this study generally did not provide evidence that altered sleep architecture is a critical factor in CFS. Future studies should further scrutinize the relationship between subjective sleep quality relative to objective polysomnographic measures.

## Background

Chronic fatigue syndrome (CFS) presents a diagnostic and management challenge. A case of CFS is defined by: 1) clinically unexplained, persistent or relapsing fatigue of at least 6 months' duration that is not the result of ongoing exertion, is not substantially alleviated by rest, and results in substantial reduction in previous levels of occupational, educational, social, or personal activities, and; 2) concurrent occurrence of at least 4 accompanying symptoms (unusual post-exertional malaise, unrefreshing sleep, significant impairment in memory/concentration, headache, muscle pain, joint pain, sore throat and tender lymph nodes) [1]. No characteristic physical signs or diagnostic laboratory abnormalities herald CFS. Thus, diagnosis depends on evaluation of self-reported symptoms and ruling out medical or psychiatric conditions that could cause the illness. Similarly, the pathophysiology of CFS remains inchoate and as yet there is no definitive treatment; rather, therapy (both pharmacologic and nonpharmacologic) is directed toward relieving symptoms and improving function [2].

Attempts to elucidate the pathophysiology of CFS must consider sleep physiology. Unrefreshing sleep is the most common of the 8 CFS-defining symptoms, reported by 88 to 95% of cases identified in population studies [3,4] and 70 to 80% of patients in clinic-based studies [5]. Most of the postulated etiologies of CFS (e.g., infection, immune and hormone perturbations) affect sleep; and, conversely, primary sleep disorders, sleep deprivation and experimental disruption of sleep produce many of the features of CFS (e.g., fatigue, impaired cognition, joint pain and stiffness) [7-10]. Indeed, untreated primary sleep disorders, such as sleep apnea and narcolepsy, preclude diagnosis of CFS [1,11].

The aforementioned issues raise a central question. Does CFS account for the accompanying sleep disturbances or does an underlying sleep abnormality result in or contribute to CFS? Studies of sleep in persons identified with CFS through tertiary care clinics have not contributed substantially to answering this question [6,12-16]. Although previous studies have consistently reported inefficient sleep and documented a varying occurrence of previously undiagnosed primary sleep disorders, they have not identified characteristic disturbances in sleep architecture or a distinctive pattern of polysomnographic abnormalities associated with CFS. As with many studies of CFS, published evaluations of sleep pathology have not uniformly applied the case definition of CFS and often lack appropriate comparison groups. People with CFS use a number of prescription and over the counter medications that affect sleep [17]; yet most studies do not mention whether or not medications have been considered. Finally, we are aware of no published studies of CFS that have utilized multiple-night polysomnography, nor are we aware of any published studies that address sleep pathology in a population-based sample of persons with CFS. Because an overwhelming majority of persons with CFS remain undiagnosed [4,18], results of studies on CFS patients identified through physician practices may not be generalizable.

The objective of this study was to describe clinical and polysomnographic sleep characteristics of persons with CFS identified from the general population of Wichita, Kansas [4], compared to non-fatigued controls matched for sex, race, age, and body mass index who were randomly selected from the same population. All study participants were admitted to a research ward in Wichita for 2 days [19]. They underwent a complete physical and psychiatric evaluation, their medications were reviewed and they completed 2-overnight polysomnographic studies and a multiple sleep latency (MSLT) evaluation. This report evaluates associations of sleep disorders and variations in sleep architecture with CFS.

## Methods

### Participants

This study adhered to U.S. Department of Health and Human Services human experimentation guidelines and received Institutional Review Board approval from the CDC and collaborating institutions. All participants gave informed consent.

Between January and July 2003, we conducted a 2-day in-hospital study of adults identified with CFS from the general population of Wichita [19]. The in-hospital study enrolled people who had participated in the 1997 through 2000 *Wichita Population-Based CFS Surveillance Study *[4]. Participants in the in-hospital study were fatigued adults with medically/psychiatrically unexplained chronic fatigue identified during the surveillance study. Fifty-eight had been diagnosed at least once with CFS and 59 had unexplained chronic fatigue that was not CFS. Controls were randomly selected from the cohort who participated throughout surveillance, who did not have medical or psychiatric exclusions, and who had not reported fatigue of at least 1-month duration; they were matched to CFS cases on sex, age, race/ethnicity, and body mass index. Upon admission to this study, subjects were reevaluated for CFS symptoms and exclusionary medical and psychiatric conditions (discussed below). The 43 who, at the time of the in-hospital study, met 1994 criteria for CFS (discussed below) comprise the cases in this report. Controls are 43 individuals who had never reported fatigue during the surveillance study, who were not fatigued at the time of this in-hospital study and who had no exclusionary medical or psychiatric condition identified at the time of study (following section). Because current classification of CFS was not completely in accord with recruitment classification, strict matching was not maintained, though cases and controls were demographically comparable. Thirty-six (84%) of the 43 with CFS and 38 (88%) of the 43 controls were women; most (40 CFS and 42 controls) were white; their mean ages were 50.6 and 50.3 years, respectively; and body mass index was 29.4 and 29.3, respectively.

### Assessment and classification of CFS

Subjects who agreed to participate were admitted to a Wichita hospital research unit for 2 days. Subjects brought all their current medications so that clinic staff could record this data and maintain medication profiles throughout the study. To identify medical conditions specified by the case definition as exclusionary for CFS [1,11], participants provided a standardized past medical history, a review of current medications, underwent a standardized physical examination, and provided blood and urine for routine analysis [1,11]. To identify psychiatric conditions exclusionary for CFS, licensed and specifically trained psychiatric interviewers administered the Diagnostic Interview Schedule for current Axis I disorders [20]. Exclusionary psychiatric illnesses specified by the case definition were current melancholic depression, current and lifetime bipolar disorder or psychosis, substance abuse within 2 years and eating disorders within 5 years. A panel of physicians and psychiatrists/psychologists reviewed this information and identified subjects with disorders exclusionary for the diagnosis of CFS. Subjects with no exclusionary conditions were considered to be CFS if they met empirically measured parameters [19] of the 1994 CFS case definition [1]. Non-fatigued controls met none of the parameters.

### Medication use

As noted, clinic staff reviewed all current (prescription and over the counter) medications that study participants were taking. Study investigators (DBR, MJD, CH, JFJ, WCR), and other Emory University Department of Psychiatry and Behavioral Sciences collaborators, reviewed all medications and classified them as affecting (inducing sleep, inhibiting sleep or with mixed effects) or not affecting sleep. Those classified as affecting sleep included analgesics (e.g., hydrocodone, Lortab, oxycodone, Propoxyphene), antidepressants (e.g., Celexa™, amitriptyline, imipramine, Lexapro™, Wellbutrin™, Effexor, Prozac™, Zoloft™, Paxil™, fluoxetine), antianxiety (Alprazolam), antihistamines (e.g., diphenhydramine, chlorpheneramine, benadryl, promethazine), decongestants (e.g., pseudoephedrine, guaifenesen), anticonvulsants (e.g., Topamax, Neurotin, clonazepam), anti-sleep phase disorder (melatonin), blood pressure controlling (e.g., Clonidine, Proamatine), antipsychotics (e.g., Seroquel, Zyprexa, Fluvoxamine), stimulants (e.g., methylphenidate, Provigil), peristaltic stimulants (Metoclopramide), and muscle relaxants (cyclobenzaprine). Medications affecting sleep were handled as a binary measure (i.e., they used or did not use one or more of those named above). Analyses took into account use of sleep affecting medications, as noted below.

### Polysomnographic and Multiple Sleep Latency Techniques

Nocturnal polysomnography and daytime multiple sleep latency testing (MSLT) were conducted in a 4-bed laboratory established at Wesley Medical Center, Wichita, KS, and consisted of polysomnography on night #1, MSLT the following day and another polysomnography on night #2. Patients were asked to arrive 3 hours before their typical bedtime on Night 1 to allow adequate time for electrode application and standard biocalibrations. "Lights out" and "lights on" time were 22:00 and 07:00, respectively. The daytime MSLT testing schedule was adjusted for other measures being collected; MSLT began at 11:00 and consisted of three additional naps at 13:00, 15:00, and 17:00.

Electrophysiological measures of wakefulness and sleep were acquired and recorded with the Flaga/Medcare N7000 digital polysomnographic system on a Windows XP platform using proprietary software (Flaga/Medcare Somnologica Studio). We employed a sampling rate of 256 Hz to allow for Fast Fourier Transform of EEG signals. Standard gold cup electrodes were employed for recording of EEG, EOG, and EMG for sleep staging and appreciation of sleep architecture. Respiration was measured with inductance plethysmography-like belts placed around the chest and abdomen. A pressure transducer, positioned in close approximation to the nares provided indices of airflow. A pulse oximeter probe was applied to either the right or left index finger, to measure arterial oxygen saturation (Sa02). Electrocardiogram (ECG) was recorded with standard snap electrodes (NeuroSupplies, Waterford, CT). The following signals were recorded: central (C_3_-A_2_//C_4_-A_1_) and occipital (O_1_-A_1_//O_2_-A_2_) EEG, left and right monopolar EOG, surface mentalis EMG, ECG (modified V3), respiratory airflow and effort and surface EMG from the right and left anterior tibialis.

The polysomnographic outcome variables used in our analyses included: total sleep time (TST) (in minutes), sleep efficiency (% of time spent in bed asleep), the percentage of TST spent in non-Rem (NREM) and REM sleep, sleep latency (in minutes) to three consecutive epochs of sleep, and REM Latency, defined as the time between the first epoch of any stage of sleep and the first epoch of REM-sleep. Brief arousals were scored following criteria set forth by the American Academy of Sleep Medicine, and the number of arousals expressed as a rate per hour of sleep adjusted for TST. Periodic leg movements both with and without accompanying arousals, were scored according to conventional criteria [22], and expressed as an index of the rate of leg movements per hour of sleep, and a separately derived index of those accompanied by an American Academy of Sleep Medicine -defined arousal [23].

Daytime sleepiness was measured with the MSLT, which has demonstrated objective sensitivity to the effects of sleep deprivation, sleep fragmentation, sleep restriction, insufficient sleep, hypersomnia, and in disease states such as sleep apnea and narcolepsy [24-26]. Multiple sleep latency tests were performed and scored according to standard guidelines with the exception that four naps were recorded at 11:00, 13:00, 15:00, and 17:00. The mean sleep latency on the MSLT was defined as the mean time from lights out to the first 30-second epoch scored as sleep. A sleep onset REM was defined as one or more epochs of REM sleep occurring within 15 minutes of the first epoch scored as sleep. Mean MSLT values of 5 or less are considered to represent pathological sleepiness, scores between 5–10 are consistent with a degree of daytime sleepiness. Scores of 10 and above are considered normative and believed to denote a lack of daytime sleepiness. Because mean values on the MSLT may adversely be affected by a spurious sleep latency on a single nap opportunity [27] possibly due to what might be perceived as stressful inter-nap activities [28], median values were also computed for each subject.

### Interpretation of polysomnography data

Polysomnography data were scored by an Emory University registered polysomnology technologist (blinded as to subjects' fatigue classification). An Emory University Department of Neurology American Board of Sleep Medicine certified physician (DBR), also blinded to the subjects' fatigue classifications, interpreted results. The polysomnology technologist manually scored each recording in 30 second epochs as wake, NREM, Stages 1–4 sleep, or rapid eye movement (REM) sleep. Criteria for scoring respiratory variables were based upon those of the Sleep Heart Health Study [21]. Briefly, apnea was scored if airflow decreased to less than or equal to 25% of the immediately preceding baseline for a period of at least 10 seconds. Hypopnea was scored if either airflow or thoracic-abdominal excursion decreased by at least 30% of baseline, for at least 10 seconds, with a concomitant reduction in SaO2 of 4% or greater. The Respiratory Distress Index (apneas + hypopneas corrected for hour of sleep) was derived from these scored events. To determine the technologist's level of reproducibility, 12 randomly selected studies were scored twice, with at least a six-week interval separating the original scoring and the repeat scoring. Comparison between original and repeat scorings with Kappa analyses yielded a Kappa coefficient of 0.88.

### Diagnostic criteria for sleep disorders exclusionary for CFS

The CFS case definition specifies sleep apnea and narcolepsy as conditions that could explain fatigue and symptoms of CFS and therefore exclude the diagnosis of CFS [1,11].

#### Obstructive sleep apnea

Obstructive sleep apnea was considered clinically significant and therefore exclusionary if an individual's Respiratory Distress Index was ≥ 30 on either night, or was between 10 and 30 and the individual had an abnormal mean sleep latency of < 5 minutes during MSLT [29].

#### Narcolepsy

Narcolepsy was diagnosed if 2 out of 4 MSLTs were positive for REM sleep and mean sleep onset during MSLT was < 5 minutes [29].

### Statistical analysis

Data were analyzed by Systat (Systat Software Inc, Richmond, CA) and SAS (SAS Institute Inc, Cary, NC). we used A 2-factor analysis of variance using PROC GLM was to measure the associations between case status and medication use (yes/no) with polysomnographic variables. Log transformed values of polysomnographic variables were used when necessary to satisfy the assumption of normally distributed outcomes. Mean values for each polysomnographic variable were adjusted for medication use using the least square method (LSMEANS). All mean values presented in this paper represent arithmetic means. We also used standard and exact logistic regression models to compute odds ratios as estimates of relative risks and 95% confidence intervals for CFS associated with dichotomous polysomnographic variables (cut-offs based on 25^th ^or 75^th ^percentiles). Measurement of clinical sleep variables included a high number of zero values. Zero-inflated Poisson Regression was used to regress case status and medication use (yes/no) on continuous values of clinical sleep variables. For this final analysis, we used SAS version 9.0 (PROC NLMIXED) and an inflation probability determined by the regressors. Analyses were also performed excluding participants taking medications that affect sleep. Estimates were unchanged when analyses excluded participants taking sleep-affecting medications. For this reason, the results presented in this report do not exclude participants taking such medications, but rather adjust for them in the analysis. We used the χ^2 ^statistic or Fisher's exact test to evaluate associations between CFS and dichotomous variables.

## Results

### Primary sleep disorders

Eleven study participants had sleep disorders exclusionary for CFS (obstructive apnea n = 8, narcolepsy n = 3). Persons with CFS had a higher frequency of exclusionary sleep disorders (8 of 43, 18.6%) than non-fatigued controls (3 of 43, 7%), but the difference was not statistically significant (p = .16). The remainder of this presentation considered the remaining 35 individuals with CFS and 40 controls.

### Sleep architecture and MSLT

There were no statistically significant differences in standard polysomnographic measurements between those with CFS and controls on either night 1 or night 2. As expected, total sleep time increased in both groups between nights 1 and 2; latency to sleep onset and to REM onset decreased in both groups; and, waking after sleep onset was less common in both groups on night 2 (data not shown). As night 1 was considered to be an adaptation night, data is shown for night 2 (Table 1). Mean values, adjusted for medication use, did not differ between participants with CFS and non-fatigued controls. Interestingly, regardless of case status, medication use was independently associated with both REM latency and Stage 1 % total sleep time. Both REM latency and Stage-1 percent of total sleep time were significantly longer in study participants who reported using any sleep medications at the time of the study (P = .02, P = .01, respectively). In addition to comparing polysomnographic measurements as continuous variables between people with CFS and non-fatigued controls, we used regular and exact logistic regression to examine possible associations. We dichotomized measurements based on 25^th ^and 75^th ^percentiles among non-fatigued controls. As with the previous analyses there were no significant differences (data not shown). Virtually identical results were obtained when analysis was restricted to subjects who did not use sleep-altering medications. Finally, evaluation of multiple sleep latency testing studies yielded similar distributions of classifications; (39% normal, 35% borderline and 26% abnormal) between CFS and controls.

### Disordered breathing and periodic leg movements during sleep

Subjects with CFS had significantly more episodes of obstructive apnea and a higher Respiratory Distress Index than did the non-fatigued controls (Table 2). Nonetheless, the difference between the groups in mean obstructive episodes per hour of total sleep was not of a magnitude recognized to have a clinical impact. All other measures of disordered breathing and periodic leg movements were not different between the two groups. Use of medications affecting sleep was independently associated with a higher rate of hypopnea and leg movements episodes per hour, after adjusting for case status (P = .03, P = .05, respectively). However, use of sleep altering medication was associated with a lower rate of obstructive apneic episodes per hour (P <.0001).

## Discussion

To our knowledge, this represents the most comprehensive polysomnographic analyses of a community sample of rigorously evaluated people with CFS and frequency-matched non-fatigued controls. There were no significant differences in rates of primary sleep disorders between CFS cases and NF controls. Thus, in spite of additional attention to methodological issues, our findings are in agreement with prior studies of CFS patients identified through clinical referral [6,12-16]. Similarly, there were no differences in any measured sleep parameters, with the exception of the frequency of obstructive apnea per hour of nighttime sleep and these differences were not clinically meaningful. While subtle breathing problems during sleep might plausibly contribute to CFS, the most striking finding of this study in fact is the *absence *of readily identifiable differences in objective, polysomnographically defined, sleep parameters between subjects with CFS and non-fatigued controls. Similarly, there were no differences between persons with CFS and non-fatigued controls with respect to daytime multiple sleep latency tests. The lack of differences in overnight sleep parameters and MSLT is in contrast to the participants' self-reported symptoms. For example, 97% of persons with CFS in this study reported unrefreshing sleep compared with 20% of controls. As noted by others, persons with CFS may suffer from an element of sleep-state misperception [30]. Future studies should further scrutinize the association between subjective sleep quality and objective polysomnographic results in persons with CFS.

Disorders of sleep were common in both CFS cases and controls in this study. Indeed primary sleep disorders that may respond to therapy were identified in 13% of the overall study population. These findings were unexpected; as the population-based nature of the study eliminated referral bias and potential participants were excluded from the study of they reported diagnosed narcolepsy or sleep apnea disorders during screening interviews. Despite this, sleep apnea and narcolepsy of clinically significant severity were identified in 11 participants, requiring their exclusion from the study. As participants with sleep disorders were not identified until polysomnographic studies were performed, it is arguable that in clinical situations, referral of subjects with unexplained fatigue to a sleep laboratory should be considered in an effort to identify disorders that may respond to intervention. In research settings case ascertainment does not usually include formal sleep studies. Thus, the potential impact of including subjects with primary sleep disorders in the CFS diagnosis should be considered when interpreting results from such studies, and when designing CFS studies. Finally, MSLTs were borderline or abnormal in 60% of subjects. This may be attributed to the occurrence of sleep disorders in our study population, or to environmentally induced sleep disruption occurring during the night preceding the MSLT.

The study has several weaknesses that should be considered while evaluating the results. While one of the largest studies identifying CFS cases and NF controls from the general population to date, the small numbers of identified subjects with current CFS may not be sufficient to identify small but biologically significant differences in sleep architecture. Second, our subjects spent two nights in the sleep lab (to allow accommodation) and (although adequate to detect primary sleep disorders) this may not produce an accurate picture of subtle nocturnal sleep behaviors. Moreover, sleep-altering medications were frequently used by both CFS cases and controls and their use was much more common among CFS cases. Some of these medications have opposite effects on sleep and we chose to lump them a sleep-altering. While we employed statistical corrections for their use, may have been inadequate to fully correct for the varied impact of the different formulations. It should nonetheless be noted that stratified analyses restricted to those without sleep-altering medications yielded similar findings compared to the total sample, although the greatly reduced numbers further limited the power of the examination. Finally, the median duration of CFS in the Wichita population was 7.3 years [4]; thus, findings in this study of prevalent cases may not be applicable to those with shorter illness duration.

## Conclusion

In conclusion, although this study evaluating associations between sleep physiology and CFS addressed the major limitations and methodological issues of previous studies, we could not confirm statistically significant associations between sleep parameters and CFS. Sleep abnormalities therefore are an unlikely contributor to the pathophysiology of CFS and the illness may include sleep-state misperception. However, 18% of persons with CFS had previously unrecognized clinically severe apnea or narcolepsy, demonstrating the importance of evaluating persons with otherwise unexplainable chronic fatigue for sleep disorders. Additional, sufficiently powered, studies with CFS cases identified from the population should be conducted.

## Abbreviations used

CDC – U.S. Centers for Disease Control and Prevention

CFS – chronic fatigue syndrome

EEG – electroencephalogram

EKG – electrocardiogram

EMG – electromyography

EOG – electrooculogram

MSLT – multiple sleep latency testing

NREM – non-REM

REM – rapid eye movement

TST – total sleep time

## Competing interests

The author(s) declare that they have no competing interests.

## Authors' contributions

WCR and CH were principal investigators of the study. WCR, CH, ERU, LSY, JFJ, and MJD designed the study protocol and supervised data collection during the study. MJD designed sleep protocols, trained sleep technicians, and supervised sleep studies. WCR and EMM were responsible for statistical analysis. DBR interpreted polysomnographic data and derived clinical sleep diagnoses. All authors contributed to interpretation of data and writing the manuscript.

## Pre-publication history

The pre-publication history for this paper can be accessed here:



## Figures and Tables

**Table 1:** Analysis of Sleep Architecture in CFS and Controls, Measured on Night 2 and Adjusted for Medication Use

	**CFS**	**NF**	
	n = 35	n = 40	
	Adjusted Mean*	Adjusted Mean*	P-Value**
**Total sleep time (min)**	400.3	407.9	0.52
**Sleep period time (min)**	453.8	457.8	0.79
**Latency to sleep onset (min)**	21.3	17.1	0.47
**REM latency (min)**	98.4	106.8	0.40
**Sleep efficiency (%)**	88.3	90.2	0.32
**Wake after onset (min)**	53.8	44.0	0.69
**Wake % Sleep Period**	11.7	9.8	0.72
**# Arousals**	105.7	110.2	0.81
**Arousal index**	15.9	16.3	0.82
**Stage 1 (% TST)**	9.6	9.5	0.79
**Stage 2 (% TST)**	48.2	50.8	0.28
**Stage 3/4 (% TST)**	19.9	17.4	0.20
**REM (% TST)**	22.3	23.3	0.98

* Mean values adjusted for medication use (yes/no)

** P-values generated using 2-factor analysis of variance

**Table 2:** Distribution of Breathing and Movement Abnormalities During Sleep

**Clinical Sleep Variable**	**CFS (n = 35)**	**NF (n = 40)**	P-value*
Hypopnea			
Mean event/hr	2.66	1.79	P = 0.24
Range	(0.00 – 16.03)	(0.00 – 14.66)	
Obstructive Apnea			
Mean event/hr	0.94	0.59	P = 0.003
Range	(0.00 – 12.11)	(0.00 – 10.70)	
Central Apnea			
Mean event/hr	0.36	0.15	P = 0.18
Range	(0.00 – 2.44)	(0.00 – 1.89)	
Mixed Apnea			
Mean event/hr	0.14	0.15	P = 0.58
Range	(0.00 – 2.28)	(0.00 – 3.15)	
Snore Index			
Mean	5.31	4.57	P = 0.95
Range	(0.00 – 27.50)	(0.00 – 34.30)	
Respiratory Distress Index			
Mean	4.11	2.69	P = 0.009
Range	(0.10 – 21.21)	(0.00 – 20.35)	
Periodic Leg Movements			
Mean events/hr	4.42	4.56	P = 0.35
Range	(0.00 – 25.74)	(0.00 – 39.37)	
Periodic Leg Movements with Arousals			
Mean events/hr	1.03	0.87	P = 0.62
Range	(0.00 – 11.57)	(0.00 = 7.09)	

*P-values were generated using zero-inflated Poisson regression, adjusting for use (yes/no) of sleep medications. Mean values represent arithmetic means. Leg movement was not measured in 9 CFS cases and 1 control.
